# Impact of Cyanide and Crude Protein Content in Host Plants on Yields and Residual Cyanide Content in Eri Silkworms (*Samia ricini* D.)

**DOI:** 10.21315/tlsr2025.36.3.11

**Published:** 2025-10-31

**Authors:** Duanpen Wongsorn, Arrisa Chueakhokkruad, Jurarat Pinwiset, Nittaya Pitiwittayakul, Benya Saenmahayak

**Affiliations:** 1 Department of Plant Science, Faculty of Agricultural Innovation and Technology, Rajamangala University of Technology Isan, Nakhon Ratchasima, 30000 Thailand; 2 Department of Animal Science, Faculty of Agricultural Innovation and Technology, Rajamangala University of Technology Isan, Nakhon Ratchasima, 30000 Thailand

**Keywords:** Eri Silkworm, Cyanide, Crude protein, Cassava Varieties, Detoxification

## Abstract

This research investigated the impact of cyanide and crude protein content in host plants on feed consumption, survival rate, yield and residual cyanide content in eri silkworms. The study utilised castor leaves from a native variety and cassava leaves from five varieties—Rayong 11, Rayong 72, Huai Bong 60, Kasetsart 50 and CMR43-08-89—which exhibited varying cyanide contents (53.66 mg/kg to 365.22 mg/kg fresh weight) and crude protein contents (17.52% to 26.04% dry matter). Eri silkworms were reared under controlled laboratory conditions (25ºC–32ºC and 65–75 %R.H.) using a Completely Randomized Design (CRD). The survival rate of eri silkworms ranged from 80.00% to 94.66% and did not differ significantly (*p* > 0.05) among the different host plant treatments. However, castor leaves were the most consumed (20.8756 g/larva), resulting in higher cocoon weight (2.5990 g), pupa weight (2.2842 g) and fecundity (435.63 eggs/female moth) compared to cassava leaves (*p* ≤ 0.05). Eri silkworms reared on Rayong 72 leaves had the lowest cocoon weight, pupa weight, shell weight and fecundity. Cyanide content analysis in eri silkworms at the late fifth instar larvae stage showed no significant difference (5.71 mg/kg to 6.33 mg/kg fresh weight, *p* > 0.05). However, the highest cyanide content was observed in pupae fed Rayong 72 leaves (14.92 mg/kg fresh weight), which significantly differed from other host plants (*p* ≤ 0.05). In summary, cyanide and crude protein content in host plant leaves influenced the feed consumption, yield and cyanide residue in eri silkworms.

**HIGHLIGHTS**

- Castor leaves (native variety) exhibited the lowest cyanide content and the highest crude protein content.
- Cassava leaves Rayong 72 variety exhibited the highest cyanide content and the lowest crude protein content.
- Eri silkworms reared on castor leaves showed the highest cocoon weight, pupa weight and fecundity, along with low cyanide residues in both larvae and pupae.
- Eri silkworms reared on Rayong 72 cassava leaves exhibited lower cocoon weight, pupa weight and fecundity, along with the highest cyanide residue in the pupae.

## INTRODUCTION

The eri silkworm (*Samia ricini* D.), part of the Saturniidae family, is a wild silk moth highly valued in the textile, food and cosmetic industries (Sirimungkararat 2012; Tamta & Mahajan 2021; Singha *et al*. 2024; Kalita *et al*. 2025). Due to its favourable characteristics, the eri silkworm can feed on various plant species, including castor leaves (*Ricinus communis* L.), Kesseru (*Heteropanax fragrans* Seem.), Tapioca (*Manihot esculenta*), Papaya (*Carica papaya*), Jatropha (*Jatropha curcas*), Barpat (*Ailanthus grandis*) and Payam (*Evodia fraxinifolia*) (Kumar & Elangovan 2010; Sirimungkararat *et al*. 2013). Eri silkworms have become increasingly popular in Thailand, especially in the northern and northeastern regions, where their cultivation is actively promoted. Farmers in these areas commonly use cassava leaves as the primary feed, favouring KU50, Rayong 72 and Huay Bong 60 varieties. Using cassava leaves for eri silkworm cultivation is an efficient way to utilise cassava fulling without producing waste. These leaves are rich in protein, contain vitamins C and A, and provide dietary fibre (Latif & Muller 2015). A significant portion of the protein in cassava leaves is linamarase, an enzyme responsible for detoxifying the cyanogenic glycosides in the plant. Cyanogenic glycosides are the most toxic compounds found in cassava. There are three forms of cyanogens in cassava: cyanogenic glycoside (95% linamarin and 5% lotaustralin), cyanohydrins and free cyanide (Montagnac *et al*. 2009a; 2009b; Ndubuisi & Chidiebere 2018). The quantity of cyanogenic glycosides in cassava varies based on the plant variety and growth conditions. For instance, water deficiency can increase hydrocyanic acid levels. Other factors influencing cyanogenic content include the plant’s age, soil properties, fertiliser use and additional factors (Ndubuisi & Chidiebere 2018; Nyaika *et al*. 2024).

Cyanogenic glycosides are relatively non-toxic by themselves. However, when plant tissues are consumed and macerated or through enzymatic hydrolysis by beta-glucosidase and the gut microflora, these cyanogenic glycosides break down to release hydrogen cyanide which is toxic to animals and humans. In humans, acute cyanide intoxication can cause symptoms such as rapid respiration, a drop in blood pressure, rapid pulse, dizziness, headache, stomach pain, vomiting, diarrhea, mental confusion, twitching and convulsions. The reported acute lethal dose of hydrogen cyanide for human ranges from 0.5 to 3.5 mg per kg of body weight. Children are especially vulnerable due to their smaller body size (Kwok 2008). Ingesting cyanide from cassava leaves can cause acute toxicity in animals, manifesting as restlessness and convulsions. A dose of hydrogen cyanide between 2.0 mg/kg to 2.3 mg/kg of body weight can be fatal for animals. Regarding the impact of cyanide on insects, Riis *et al*. (2003) reported that the beetle *Cyrtomenus* bergi laid fewer eggs when feeding on cassava roots containing cyanogenic levels exceeding 150 ppm (expressed as hydrogen cyanide) on a fresh weight basis (or 400 ppm on a dry weight basis).

The hypothesis suggests that eri silkworms reared on host plants with high nutritional value and low cyanide content will demonstrate enhanced feed consumption and improved commercial traits, while accumulating lower levels of cyanide residues. Hence, examining the influence of cyanide present in castor and cassava leaves on eri silkworms’ cultivation is vital for comprehending the impact of different cyanide levels on feed consumption and survival rate. This knowledge provides essential insights for selecting cassava varieties that are conducive to cultivating and enhancing eri silkworm yield in the future.

## MATERIALS AND METHODS

### Host Plant Cultivation

Castor leaves from a native variety and cassava leaves from various cultivars (Rayong 11, Rayong 72, Huay Bong 60, Kasetsart 50 (KU50) and CMR43-08-89), commonly cultivated in Nakhon Ratchasima province, were selected for this study. Castor seeds and cassava stem cuttings were planted in the experimental agricultural plot at Nongrawiang Educational Center, Rajamangala University of Technology Isan, Nakhon Ratchasima, Thailand. One month after germination, NPK fertiliser (15–15–15) was applied at a rate of 30 kg per rai, following the guidelines of the Field and Renewable Energy Crops Research Institute (2020). The plants were used for the experiment once they reached six months after planting.

### Cyanide and Protein Content Analysis in Host Plants

Host plant leaves were randomly selected from the cultivation field six months after planting. Sampling was conducted between 06:00 a.m. and 07:00 a.m. From each of the 15 plants per cultivar, 15 healthy leaves were collected from the upper and middle stem sections, within 30 cm of the apex. Then, cyanide level was analysed using a method modified from Surleva *et al*. (2013), with the following procedures: the host plant leaves were ground and homogenised in a pestle and mortar. Then, 10 mg of the obtained powder was transferred into a test tube, where 5 mL of 0.1% NaHCO_3_ was added. The sample was then sonicated for 20 min in a water bath. Next, 1 mL of the mixture was centrifuged at 10,000 rpm for 10 min. Two aliquots of supernatant (40 μL each) were added to 1 mL of 2% Na_2_CO_3_ and 0.5 mL of ninhydrin solution. The mixture was homogenised and incubated for 15 min for colour development. The absorbance of the samples was recorded at 485 nm.

The method for crude protein analysis was adapted from Priyadarshini and Revanasiddaiah (2013), with the following steps: host plant samples of cassava leaves were subsampled and freshly weighed at the time of collection. They were then oven-dried at 65°C until they reached a constant weight to determine dry matter (DM) and moisture content. The host plant leaves moisture content was measured by taking fresh and dry weight of the leaves (Zhu *et al*. 2020).


Moisture content (%)=[(Fresh weight-Dry weight)×100]/Fresh weight

The dried samples were ground with a hammer mill through a 1-mm sieve and stored for subsequent chemical and *in vitro* analyses. Crude protein (Kjeldahl method) was analysed according to the standard methods of Association of Official Analytical Chemists (AOAC 2000).

Eri silkworm eggs of the Kamphaeng Saen variety were supplied by the Center of Excellence for Sericulture, Faculty of Agriculture, Kasetsart University, Kamphaeng Saen Campus. The eggs were incubated for hatching under controlled conditions of 25°C–32°C and 65%–75% relative humidity in the Plant Science Laboratory at Rajamangala University of Technology Isan, Nakhon Ratchasima Campus.

Eri silkworms in their first instar were divided and placed in insect rearing boxes measuring 6.3 × 9.6 × 3.5 inches after they hatched simultaneously over a span of 3 h to 5 h. They were then given various host plants (castor and cassava leaves from Rayong 11, Rayong 72, Huay Bong 60, Kasetsart 50 and CMR43-08-89 cultivars). The experiment followed a Completely Randomized Design (CRD) with three replicates for each type of host plant. Each replicate consisted of 150 larvae fed ad libitum twice a day with equal-quality host plants across all treatments. Before each feeding, plant leaves were weighed, and any remaining leaves were collected to calculate the amount of feed consumed at each instar. The rearing process followed the method described by Wongsorn *et al*. (2024) and conducted in accordance with standard animal research protocols (Animal Use License No. U1-02971-2559).

Throughout the experiment, comprehensive data on the rearing performance of eri silkworms were recorded. These data included survival rate, cocoon weight, pupa weight, shell weight, shell ratio, fecundity and hatchability.

Survival rate of larvae (%): This was calculated by dividing the number of surviving larvae by the total number of larvae hatched, expressed as percentage.

Cocoon weight (g): On day 7 of spinning, 10 cocoons were randomly selected and harvested from each replication. The weight of each cocoon was measured and their average was recorded.

Shell weight (g): Ten cocoons were randomly selected and cut open, and their pupae and larval excuvium were removed. The average shell weight was then recorded separately.

Pupal weight (g): Similarly, following the opening of the cocoons, the weight of a single pupa was recorded using a sensitive balance. This can also be determined by subtracting the shell and exuviae weight from the cocoon weight.

Shell ratio (%): The quantity of silk within a cocoon shell was expressed as a percentage, calculated as the cocoon shell’s weight divided by the cocoon’s weight with pupa.

Fecundity (eggs/moth): Pairs of freshly emerged moths were hung on a wire and placed on a montage. Each treatment had three replications, with five pairs of moths in each replication. After three hours, the moths were decoupled, and female moths were allowed to lay eggs on the montage. Three days later, the eggs were separated from the montage and counted replication-wise to determine the fecundity, recorded as the number of eggs per female.

Hatchability (%): Hatchability was determined by subtracting the number of non-hatched eggs from the total number of laid eggs and then dividing by the number of normal eggs.

### Cyanide Content Analysis in Eri Silkworm

At the end of the 5^th^ instar (mature) stage, eri silkworms were randomly selected and allowed to spin cocoons. Eri silkworm pupae, 7 days old and weighing 150 g each, were randomly selected. The pupae were then boiled in water at approximately 100°C for 10 min. Then, cyanide content was analysed using the cyanide method modified from Surleva *et al*. (2013).

Statistical analysis: The data were subjected to analysis of variance (ANOVA) using SAS programme version 9.00 (SAS Institute Inc. 2006). Significant differences between treatment means were delineated and determined using Duncan’s New Multiple Range Test (DMRT) at a 5% level of significance.

## RESULTS

### Cyanide and Protein Content in Host Plants

The cyanide content in the leaves of host plants is summarised in Table 1. Among the cassava leaf varieties, Rayong 72 exhibited the highest cyanide content (397.89 mg/kg fresh weight), followed by KU50 (365.22 mg/kg fresh weight) and Huay Bong 60 (311.10 mg/kg fresh weight), with no statistically significant difference (*p* > 0.05). However, there was a statistically significant difference (*p* ≤ 0.05) between CMR43-08-89 (142.21 mg/kg fresh weight) and Rayong 11 (105.49 mg/kg fresh weight), which had lower cyanide content. The variety with the lowest cyanide content was castor leaves at 53.66 mg/kg fresh weight.

The highest crude protein content observed in castor leaves was 26.04% DM, which was significantly higher (*p* ≤ 0.05) than that in cassava leaves. Rayong 11, Rayong 72, Huay Bong 60, KU50 and CMR43-08-89 contained crude protein content of 17.85, 17.52, 19.31, 21.05 and 21.60 %DM, respectively. The moisture content of castor leaves (70.07%) and KU50 variety (69.37%) was comparable, with no statistically significant difference observed between them (*p* ≤ 0.05). However, both values differed significantly (*p* ≤ 0.05) from the other cassava varieties.

### Feed Consumption of Eri Silkworm

The feed consumption of eri silkworms is shown in Table 2. The eri silkworms consumed the highest amount of castor leaves during stages 1^st^–5^th^ instar, averaging 20.8756 g/larva. The consumption differed significantly (p ≤ 0.05) from that of the cassava plants. Cassava leaves of the CMR43-08-89 variety were consumed at an average of 14.8631 g/larva, while Rayong 11 was consumed at an average of 14.0351 g/larva. Conversely, Rayong 72 exhibited the lowest consumption at 13.0656 g/larva.

### Survival Rate of Eri Silkworm

From the 1^st^ to the 4^th^ instar, the eri silkworm exhibited a 100% survival when fed with all host plants (Table 3). In the 5^th^ instar, silkworms reared on cassava leaves, particularly the Rayong 11 and Rayong 72 varieties showed a survival rate of 99.33%, which was not significantly different (*p* > 0.05) from those fed on other host plants. Across the entire larval to adult stage, survival rates ranged from 80.00% to 94.66%, with no significant differences observed (*p* > 0.05).

### Cocoon and Eggs Yield Parameters of Eri Silkworm

The eri silkworms reared on castor leaves had the highest yields of cocoon weight, pupa weight and fecundity with values of 2.5990 g, 2.2842 g and 435.63 eggs, respectively (Table 4). These results showed statistically significant differences (*p* ≤ 0.05) compared to those reared on cassava leaves. However, no statistically significant differences (*p* > 0.05) were observed in shell weight, shell ratio, and hatchability across all treatments. The shell weight ranged from 0.2243 to 0.3030 g, shell ratio from 11.16% to 14.45%, and hatchability from 88.74% to 91.05%.

### Cyanide Content in 5^th^ Instar Larvae and Pupae of Eri Silkworm

**FIGURE 1:**
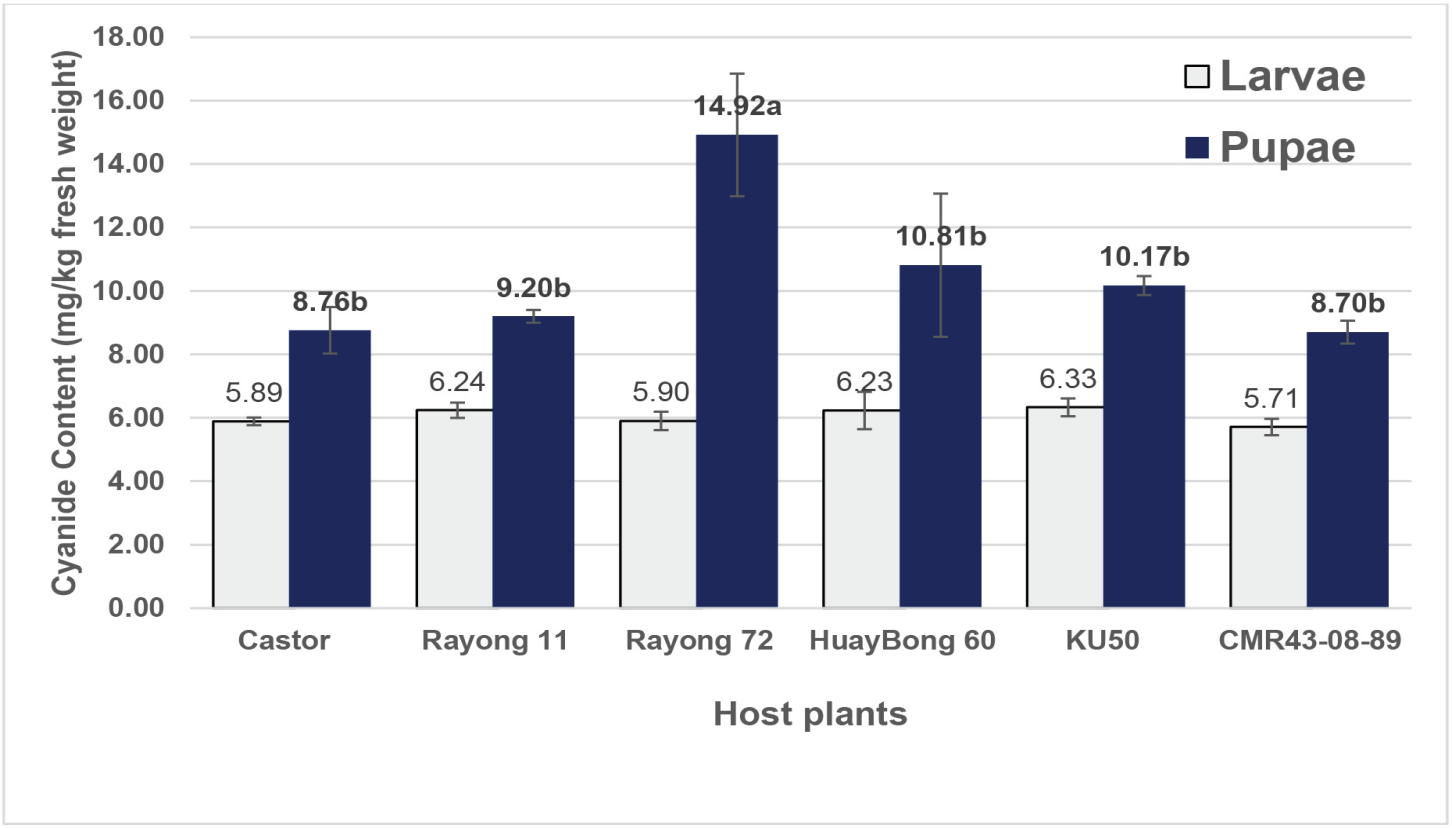
Cyanide content of larvae and pupae of eri silkworm (*Samia ricini* D.) reared on different host plants. (Means with the same letter were not significantly different (*p* > 0.05) according to DMRT).

When reared on different host plants, the cyanide content of eri silkworm larvae at the final 5^th^ did not show significant differences (*p* > 0.05) (Fig. 1). The cyanide content, ranked from lowest to highest, was as follows: CMR43-08-89, Castor, Rayong 72, Huay Bong 60, Rayong 11 and KU50, with values of 5.71, 5.89, 5.90, 6.23, 6.24 and 6.33 mg/kg fresh weight, respectively. The highest cyanide content in the pupae was found in eri silkworm reared on Rayong 72 cassava leaves, at 14.92 mg/kg fresh weight, which was statistically different (*p* ≤ 0.05) from eri silkworm reared on other host plants. Following this were eri silkworm reared on Huay Bong 60 (10.81 mg/Kg fresh weight), while eri silkworm reared on CMR43-08-89 had the lowest cyanide content at 8.70 mg/Kg fresh weight, similar to those reared on castor leaves.

## DISCUSSION

This study revealed that the tested host plants contained varying levels of cyanide, crude protein and moisture content. Similar to the findings of Jamila and Bujang (2016), who reported that cassava leaves from different varieties had varying nutrient compositions, including crude protein, crude lipid, crude fibre, ash, carbohydrate and antinutrient contents (cyanide and tannin). Obueh and Kolawole (2016) also observed that the sweet cassava variety contained higher levels of moisture, crude protein, fat, crude fibre and ash compared to the bitter cassava variety. Furthermore, the sweet cassava variety contains up to three times less hydrogen cyanide than the bitter cassava variety, indicating that the sweet cassava variety is a good source of lipids, nitrogenous compounds and cellulose. Similarly, variations in biochemical factors (leaf moisture, chlorophyll, crude protein and total carbohydrates) and macronutrients (nitrogen, phosphorus, potassium, calcium, magnesium and sulfur) have been observed among different castor genotypes (Chandrashekhar *et al*. 2013). The nutrient composition in leaves is influenced by genetic, physiological, edaphic and climatic variations, as well as by maturity stage, which is the primary factor contributing to nutrient diversity within leaves (Ravindran 1995; Mossie *et al*. 2024).

Feeding eri silkworms with host plants that have varying crude protein and cyanide content affects their feed intake differently. Eri silkworms consumed castor leaves the most, likely due to the higher crude protein and moisture content of castor leaves compared to cassava leaves. Additionally, the succulence and tenderness of castor leaves allowed eri silkworms to consume more. This is consistent with the findings of Shifa *et al*. (2014), who reported that eri silkworms fed with different genotypes of castor leaves exhibited varying feed intake and growth rates. Moreover, the nutrient absorption and growth rate of eri silkworms also differed depending on plant food varieties and genotypes (Chhatria *et al*. 2017).

However, this study found that the crude protein and cyanide content did not affect the survival of the eri silkworm. Instead, these factors influenced the commercial traits of the silkworm. Castor leaves with higher crude protein and lower cyanide levels resulted in significantly better values for parameters, such as fresh cocoon weight, fresh pupae weight and number of egg/moth, compared to those fed with cassava leaves containing lower crude protein and higher cyanide levels. According to Sakthivel (2016), the MVD1 variety of cassava leaves contained the highest levels of essential nutrients, including protein, total carbohydrates, nitrogen, phosphorus, potassium and total minerals, along with the lowest levels of antinutrients such as tannins and HCN. In contrast, the CO_2_ variety had lower levels of essential nutrients and higher levels of antinutrients. When rearing eri silkworm, MVD1 exhibited superior economic traits, while CO_2_ was identified as a poor performer, closely followed by H226, with CO_2_ showing the least favorable values. Derara *et al*. (2020) observed that cassava varieties and nitrogen and mineral application rates significantly influenced eri silkworm grainage, larval development and cocoon yield parameters. Additionally, Thanga *et al*. (2021) reported that rearing eri silkworms on castor leaves with high moisture content, protein, and total carbohydrates led to superior economic traits, including cocoon yield and silk percentage as indicated by mature larval weight, single cocoon weight, shell weight, shell ratio and effective rate of rearing. The quality of the leaves directly influenced the eri silkworm. Plants with high nutritional values (crude protein, crude fat, carbohydrates, vitamins and amino acids) and moisture content play a major role in the health, growth, survival and cocoon parameters of eri silkworms (Singha *et al*. 2015; Mossie *et al*. 2024).

Regarding cyanide content, the eri silkworm larvae had lower cyanide content compared to pupae. This difference can be attributed to the activity of *β*-cyanoalanine synthase during the larval stage, which detoxifies cyanide. According to Witthohn and Naumann (1987), *β*-cyanoalanine synthase activity is exclusively present during the larval feeding stages of the butterfly H. melpomone (Papilionoidea), aligning with the developmental phases where detoxification of ingested cyanogenic plant material is necessary. In butterflies and moths, *β*-cyanoalanine synthase predominantly handles the detoxification of hydrogen cyanide produced from the breakdown of cyanogenic glucosides, while other arthropods rely on the enzyme rhodanese (Zagrobelny *et al*. 2018).

The statistical analysis showed no significant differences in cyanide levels among eri silkworm larvae fed different host plants. However, the cyanide levels in pupae corresponded to those found in cassava leaves. For instance, cassava leaves from Rayong 72 variety had the highest cyanide content, leading to the highest cyanide levels in eri silkworm as well. This is consistent with Maskhao *et al*. (2017), who reported that eri silkworms reared on cassava leaves had lower cyanide levels than their pupae, and the cassava leaves of the Rayong 72 variety had higher cyanide levels compared to other varieties (Five minutes, Huay Bong 80, Rayong 72, Rayong 9 and Kasetsart 50). According to Hay-Roe (2004), the variation in cyanogenic compound levels among Heliconius larvae could stem from differences in the cyanogenic substances present in the host plants and from the larvae’s detoxification mechanisms. Furthermore, cyanide concentrations varied at different developmental stages of the insect. The highest concentrations of cyanide-releasing compounds occur in the egg stage, with no significant differences in concentration between the larval and pupal stages. Nevertheless, cyanogenic compound concentrations increased in adults due to cyanoglucoside biosynthesis during this stage (Nahrstedt & Davis 1985). Differences in strategies for coping with cyanogenic compounds likely stem from genetic variation among insect races. Some butterflies and moths possess mechanisms to break down cyanogenic glucosides, primarily detoxifying the released hydrogen cyanide through *β*-cyanoalanine synthase. In contrast, other arthropods utilize the enzyme rhodanese for detoxification (Hay-Roe & Nation 2007; Zagrobelny *et al*. 2018). According to Igue and Agboola (2013), the survival of grasshopper (*Zonocerus variegatus*), which feeds on cyanogenic plants such as cassava, depends on the presence of the enzyme rhodanese. This enzyme demonstrated significant activity and possessed appropriate kinetic properties in the grasshopper’s gut, particularly when the grasshopper predominantly consumed cyanogenic cassava leaves.

Based on previous reports, Rhodanese and *β*-cyanoalanine synthase—key enzymes involved in cyanide detoxification—are active in both the larval and adult stages of insects (Beesley et al. 1985). Antony *et al*. (2006) reported increased activity of these enzymes in Bemisia tabaci when fed on cyanogenic cassava compared to non-cyanogenic sweet potato. Consequently, future investigations should aim to assess the concentration and functional efficiency of detoxification enzymes in eri silkworms reared on various host plant species, to elucidate potential metabolic adaptations to dietary cyanide.

## CONCLUSIONS

Castor leaves had the highest crude protein content and the lowest cyanide content. This resulted in the highest cocoon weight, pupa weight and fecundity in the eri silkworms, as well as low cyanide residue in both larvae and pupae. On the other hand, Rayong 72 cassava leaves had the lowest crude protein content and the highest cyanide content. Consequently, eri silkworms reared on Rayong 72 cassava leaves exhibited lower cocoon weight, pupa weight and fecundity along with the highest cyanide residue in the pupae. This indicated that both crude protein and cyanide content significantly affected the productivity of eri silkworms and the cyanide residue in their pupae.

## Figures and Tables

**TABLE 1:** Cyanide, crude protein and moisture contents of different host plants used for eri silkworm rearing.

Host plants	Cyanide content (mg/kg fresh weight ± SD)	Crude protein content (%DM ± SD)	Moisture content (% ± SD)
Castor	53.66 ± 0.84 b	26.04 ± 0.27 a	70.07 ± 1.66 a
Rayong 11	105.49 ± 14.61 b	17.85 ± 0.19 d	66.88 ± 0.67 b
Rayong 72	397.89 ± 82.69 a	17.52 ± 0.58 d	66.88 ± 0.67 b
Huay Bong 60	311.10 ± 42.90 a	19.31 ± 0.44 c	66.27 ± 0.25 b
KU50	365.22 ± 42.93 a	21.05 ± 0.25 b	69.37 ± 1.75 a
CMR43-08-89	142.21 ± 54.31 b	21.60 ± 0.29 b	67.56 ± 1.12 b
*p*-value	< 0.0001	< 0.0001	0.0005

*Notes:* Means ± SD followed by the same letter within a column are not significantly different (DMRT, *p* > 0.05); SD = standard deviation

**TABLE 2:** Feed intake of eri silkworms (*Samia ricini* D.) reared on different host plants.

Host plants	Food intake (g/larva ± SD)					

	1^st^ instar	2^nd^ instar	3^rd^ instar	4^th^ instar	5^th^ instar	1^st^–5^th^ instar
Castor	0.0554 ± 0.00 a	0.1500 ± 0.00 ab	0.9324 ± 0.02 a	2.3373 ± 0.01 a	17.4004 ± 0.64 a	20.8756 ± 0.61 a
Rayong 11	0.0384 ± 0.00 a	0.1461 ± 0.00 b	0.8539 ± 0.01 b	2.2883 ± 0.08 ab	10.7082 ± 0.56 bc	14.0351 ± 0.63 bc
Rayong 72	0.0337 ± 0.00 b	0.1452 ± 0.00 b	0.7368 ± 0.04 c	2.1271 ± 0.15 b	10.0227 ± 0.93 c	13.0656 ± 1.03 c
Huay Bong 60	0.0256 ± 0.00 c	0.1447 ± 0.00 b	0.4427 ± 0.02 d	1.6706 ± 0.06 c	11.0127 ± 0.41 bc	13.2964 ± 0.47 c
KU50	0.0372 ± 0.00 b	0.1528 ± 0.00 a	0.7428 ± 0.00 c	2.1457 ± 0.10 b	10.3108 ± 0.52 c	13.3894 ± 0.39 c
CMR43-08-89	0.0383 ± 0.00 b	0.1440 ± 0.00 b	0.7637 ± 0.03 c	2.3339 ± 0.04 a	11.5830 ± 0.12 b	14.8631 ± 0.08 b
*p*-value	< 0.0001	0.0365	< 0.0001	< 0.0001	< 0.0001	< 0.0001

*Notes:* Means ± SD followed by the same letter within a column are not significantly different (DMRT, *p* >0.05); SD = Standard deviation.

**TABLE 3:** Survival rate of eri silkworms (Samia ricini D.) reared on different host plants.

Host plants	Survival (% ± SD)					

	Larva stage (1^st^–5^th^ instar)					Larva - adult stage

	1^st^ instar	2^nd^ instar	3^rd^ instar	4^th^ instar	5^th^ instar	
Castor	100.00 ± 0.00	100.00 ± 0.00	100.00 ± 0.00	100.00 ± 0.00	100.00 ± 0.00	94.66 ± 4.16
Rayong 11	100.00 ± 0.00	100.00 ± 0.00	100.00 ± 0.00	100.00 ± 0.00	99.33 ± 1.15	88.00 ± 3.46
Rayong 72	100.00 ± 0.00	100.00 ± 0.00	100.00 ± 0.00	100.00 ± 0.00	99.33 ± 1.15	80.00 ± 12.16
Huay Bong 60	100.00 ± 0.00	100.00 ± 0.00	100.00 ± 0.00	100.00 ± 0.00	100.00 ± 0.00	82.66 ± 8.08
KU50	100.00 ± 0.00	100.00 ± 0.00	100.00 ± 0.00	100.00 ± 0.00	100.00 ± 0.00	86.00 ± 10.00
CMR43-08-89	100.00 ± 0.00	100.00 ± 0.00	100.00 ± 0.00	100.00 ± 0.00	100.00 ± 0.00	94.00 ± 5.29
*p*-value	-	-	-	-	0.5705	0.2076

*Notes:* Means ± SD followed by the same letter within a column are not significantly different (DMRT, *p* > 0.05); SD = Standard deviation.

**TABLE 4:** Commercial traits of eri silkworm (*Samia ricini* D.) reared on different host plants.

Host plants	Parameter					

	Cocoon weight (g ± SD)	Pupa weight (g ± SD)	Shell weight (g ± SD)	Shell ratio (% ± SD)	Fecundity (eggs/female moth ± SD)	Hatchability (% ± SD)
Castor	2.5990 ± 0.13 a	2.2842 ± 0.10 a	0.3030 ± 0.03	11.65 ± 0.15	435.63 ± 50.75 a	90.32 ± 0.74
Rayong 11	2.0631 ± 0.08 bc	1.8287 ± 0.07 bc	0.2303 ± 0.02	11.16 ± 0.52	305.36 ± 8.31 c	90.24 ± 1.23
Rayong 72	1.8975 ± 0.02 d	1.6622 ± 0.02 d	0.224 3± 0.01	11.82 ± 0.37	254.43 ± 12.35 d	91.05 ± 4.39
Huay Bong 60	1.9741 ± 0.06 cd	1.7242 ± 0.05 cd	0.2377 ± 0.01	12.08 ± 0.38	290.70 ± 5.96 cd	88.74 ± 5.19
KU50	2.1471 ± 0.04 b	1.8759 ± 0.03 b	0.3103 ± 0.08	14.45 ± 3.45	353.40 ± 34.19 b	89.49 ± 3.69
CMR43-08-89	2.0619 ± 0.12 bc	1.8004 ±0.11 bc	0.2515 ± 0.01	12.20 ± 0.56	319.53 ± 3.87 bc	90.24 ± 2.75
*p*-value	< 0.0001	< 0.0001	0.0545	0.2188	< 0.0001	0.9711

*Notes:* Means ± SD followed by the same letter within a column are not significantly different (DMRT, *p* > 0.05); SD = standard deviation.
